# Microbial Communities in Volcanic Glacier Ecosystems

**DOI:** 10.3389/fmicb.2022.825632

**Published:** 2022-04-25

**Authors:** Eva Garcia-Lopez, Fatima Ruiz-Blas, Silvia Sanchez-Casanova, Sonia Peña Perez, Maria Luisa Martin-Cerezo, Cristina Cid

**Affiliations:** ^1^Department of Molecular Evolution, Centro de Astrobiologia (CSIC-INTA), Torrejón de Ardoz, Spain; ^2^IdiPAZ, Hospital Universitario La Paz, Madrid, Spain; ^3^IFM Biology, Linköping University, Linköping, Sweden

**Keywords:** bacteria, glacier, volcano, microbial communities, ecology

## Abstract

Glaciers constitute a polyextremophilic environment characterized by low temperatures, high solar radiation, a lack of nutrients, and low water availability. However, glaciers located in volcanic regions have special characteristics, since the volcanic foci provide them with heat and nutrients that allow the growth of microbial communities highly adapted to this environment. Most of the studies on these glacial ecosystems have been carried out in volcanic environments in the northern hemisphere, including Iceland and the Pacific Northwest. To better know, the microbial diversity of the underexplored glacial ecosystems and to check what their specific characteristics were, we studied the structure of bacterial communities living in volcanic glaciers in Deception Island, Antarctica, and in the Kamchatka peninsula. In addition to geographic coordinates, many other glacier environmental factors (like volcanic activity, altitude, temperature, pH, or ice chemical composition) that can influence the diversity and distribution of microbial communities were considered in this study. Finally, using their taxonomic assignments, an attempt was made to compare how different or similar are the biogeochemical cycles in which these microbiomes are involved.

## Introduction

Volcanic activity is, together with climatic changes, one of the environmental factors that most influence the behavior of glaciers (Barr et al., 2018). These changes typically occur either because glaciers are located on active volcanoes, or because volcanic products interact with glaciers in adjacent regions. So far it has been thoroughly studied, from a geological point of view, how volcanic activity affects glacial behavior (Kirkbride and Dugmore, 2003). Even volcanism and ice interactions have drawn a focus on glacial and volcanic activity on Mars (Chapman et al., 2000; Scanlon et al., 2014). However, the effect on the microbial populations that inhabit these glaciers has not been studied in detail (Garcia-Lopez et al., 2016). Glaciers constitute a polyextremophilic environment characterized by low temperatures, high solar radiation, a lack of nutrients and, low water availability (Anesio et al., 2017; Hotaling et al., 2017). However, glaciers located in volcanic regions have special characteristics, since explosive eruptions create a transient bridge between the solid earth and the atmosphere, frequently injecting volcanic aerosols that contribute to the dispersion of microorganisms (Van Eaton et al., 2013). Besides, volcanic foci provide them with heat and nutrients. Pyroclastic materials and gases emitted by volcanoes constitute an important source of nutrients for the microorganisms that inhabit these glaciers (Fazlutdinova et al., 2021). In turn, microbial activity plays a fundamental role in the degradation and chemical exchange between rocks and meltwater.

It is well-known that glaciers present diverse horizontal strata with different environmental characteristics (i.e., temperature, solar radiation, and nutrient content; Hodson et al., 2008; Lanoil et al., 2009; Boetius et al., 2015). These factors determine the biodiversity of the microbial communities that inhabit them (Edwards et al., 2011; Garcia-Lopez and Cid, 2017). The glacier basal bedrock and subglacial lakes constitute a subglacial ecosystem populated by aerobic chemoheterotrophs and by anaerobic methanogens, nitrate reducers, and sulfate reducers (Skidmore et al., 2000; Phillips and Parnell, 2006; Mikucki et al., 2009; Harrold et al., 2016). Most of the microbiological research that has been carried out to date in the subglacial ecosystem has focused on glaciers with a granite or carbonate bedrock, but the processes that support microbial life in subglacial systems located on volcanic soils are not well understood (Hamilton and Havig, 2017). Furthermore, most of the studies on these microbial communities have been carried out in volcanic environments in the northern hemisphere, mainly in Iceland (Gaidos et al., 2004; Lutz et al., 2015) and in the Pacific Northwest (Brown et al., 2016; Hamilton and Havig, 2017). The northern hemisphere has more terrestrial volcanic glaciers than the southern hemisphere due to plate tectonics, and this is a significant north–south difference (Russell et al., 2014; Yeh and Shellnutt, 2016).

To get to know the microbial diversity of the underexplored southern subglacial ecosystems and to check what their specific characteristics were, we researched the microbiomes of volcanic glaciers located around parallels 52 north and 60 south. Specifically, the volcanoes were located on Deception Island, in Antarctica, and on the Kamchatka Peninsula, in Russia. In this study, we conducted an analytical approach that combines geochemical assays, molecular (high throughput rRNA gene sequencing)-based techniques, and several multivariate statistical analyses to answer some appealing questions: (i) what are the microorganisms that inhabit these volcanic glaciers?; (ii) what are the similarities and differences in the microbial populations of volcanic glaciers in terms of relative abundance and diversity?; and (iii) how is their ecology and distribution concerning environmental parameters (volcanic activity, latitude, temperature, and ice chemical composition)? To answer these questions, the microbiomes of four volcanic glaciers from the Antarctica (Macaroni and Kirkwood) and Kamchatka (Gorely and Mutnovsky) were researched.

## Materials and Methods

### Field Site Descriptions, Sample Collection, and Processing

**Figure 1:**
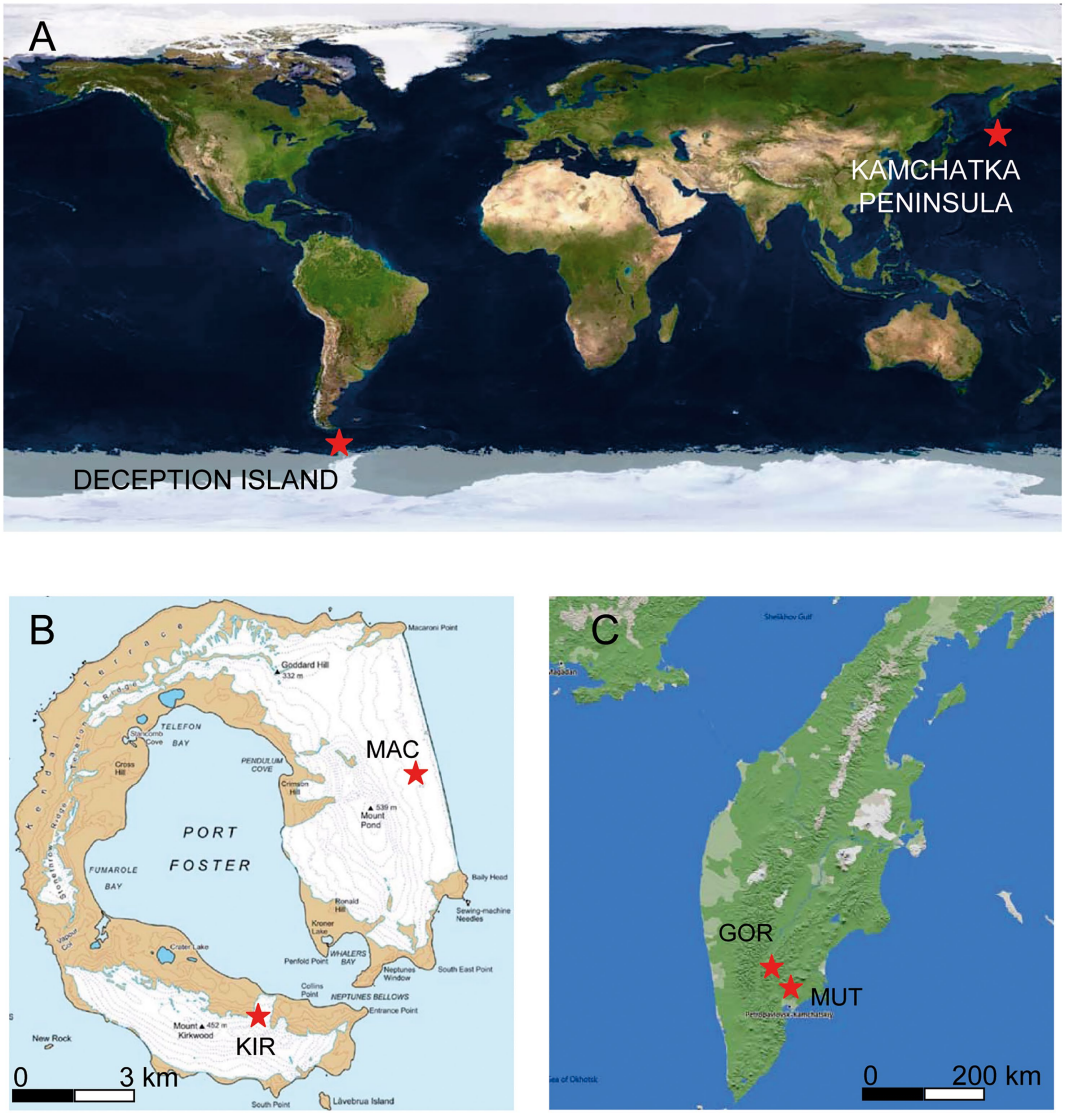
Maps of the sample point locations. **(A)** Overview map. **(B)** Map of Deception Island. **(C)** Map of the Kamchatka Peninsula. Map image source: Google Earth Pro.

**Figure 2:**
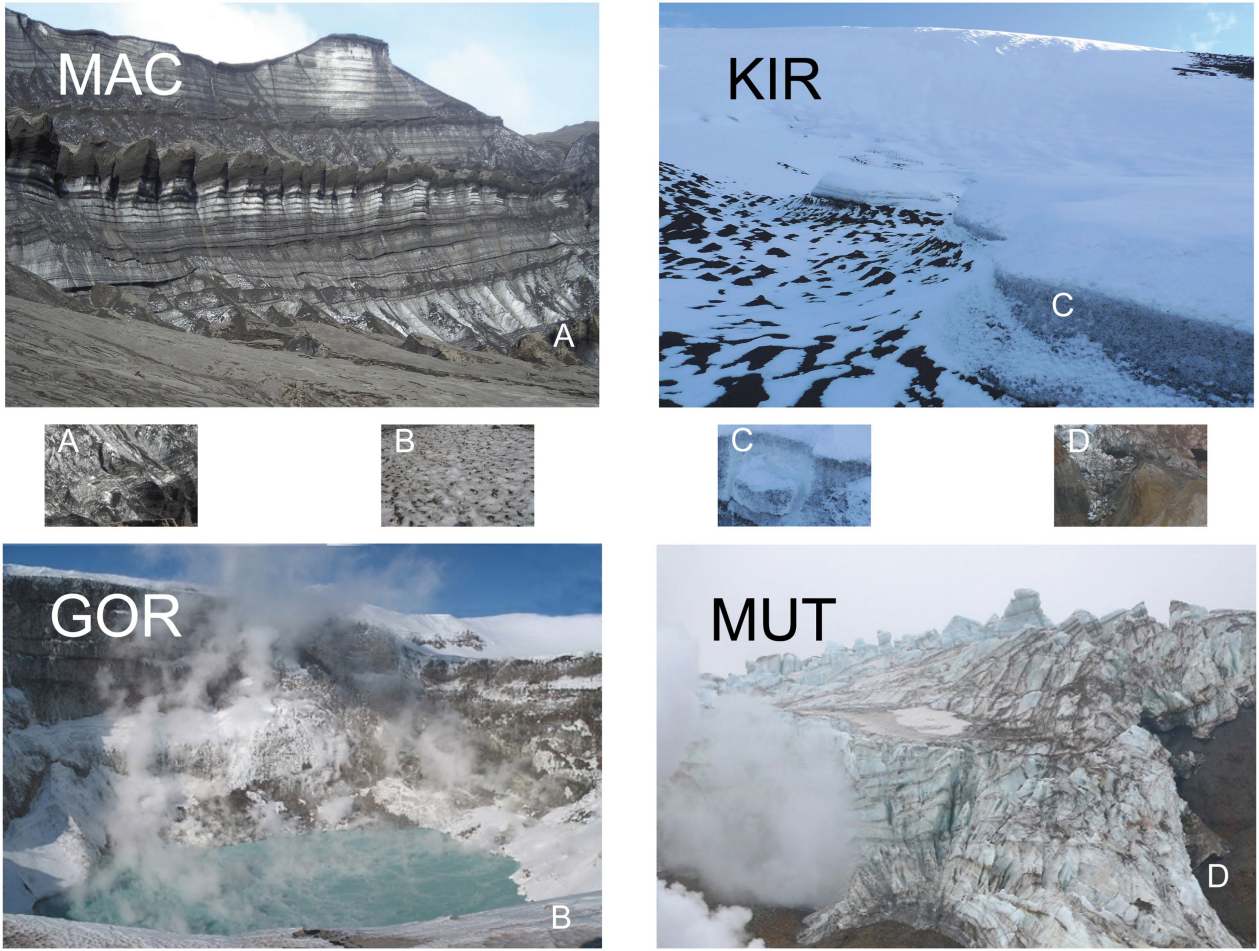
Pictures of volcanic glaciers. Pictures of glaciers MAC, KIR, GOR, and MUT. **(A–D)** represent the sampling sites.

Deception Island is a volcanic horseshoe-shaped island 14 km north–south by 13 km east–west (Figure 1). It reaches a height of 542 m at the summit of Mount Pond and 459 m on Mount Kirkwood, both of which have a permanent glacier cover (Baker et al., 1975). From Mount Pond to the east, there is a cliff of more than 100 m in height that culminates in the Macaroni Point. The center of the island is a large caldera that communicates with the open sea on its southeast slope. The most recent recorded volcanic eruptions in Deception Island took place in the years 1842, 1967, 1969, and 1970 (Wilkes, 1845; Orheim, 1971). Two main types of volcanic rocks appeared in the form of pyroclast cones and lava flows, depending on whether the vent was wet or dry at the time of the eruption. The pyroclast cones were essentially built where water had access to the rising magma. This is what happened in the 1969 eruption, which was a subglacial eruption, resulting in pyroclast cones consisting of dark brown lapilli and ash (basaltic andesite) embedded in alternating layers of glacial ice around Macaroni Point (Figure 2; Gonzalez-Ferran and Katsui, 1970). In contrast, on the slopes of Mount Kirkwood, the vent was dry and the pyroclastic material had the form of lava flows (basalt and basaltic andesite), which were later encased in glacial ice (Figure 2; Hawkes, 1961; Clapperton, 1969).

The volcanoes located on the Kamchatka peninsula are part of the Pacific Ring of Fire (between 58°N and 55°N; Figure 1). Its Middle Range is studded with numerous extinct and dormant volcanoes, of which about 30 are currently active. Active volcanism and tectonics characterize the area and are the main factors shaping the landscape (Ivanov, 2002). Some of the volcanic cones reach a considerable height and many support snowfields and glaciers. In this work, we focus on two of them: Gorely and Mutnovsky. The climate of this region is influenced by its geographical position and by the cold East Kamchatka Current. Although the climate is considered sub-Arctic, in recent years the winters are relatively mild and at present, there is no permafrost south of 57°N (Jones and Solomina, 2015). The Mutnovsky volcano is in a state of constant gaseous activity (Figure 2). It consists of a caldera filled by a glacier that contains clear ice interbedded with pyroclastic materials. An ice-fall within the caldera is covered by volcanic sand and blocks of lava. The lavas are basaltic, andesitic, and dacitic. A large proportion of them is highly decomposed and impregnated with sulfur and gypsum (Tomkeieff, 1949). The Gorely is an active volcano with intense eruptions and gas emissions (Figure 2). Its top is framed by a chain of explosive craters, and its large caldera is partly obliterated by numerous lava flows composed of augite-andesites (Kuznetsov et al., 2017).

Ice for DNA extraction was collected in triplicate from four glaciers that override volcanic terrains; two of them are located in the Kamchatka Peninsula, and two of them are in Deception Island, South Shetland Archipelago, and Antarctica (Table 1). Glacial ice samples were cored in summer (August 2015) and in austral summer (January 2015).

**Table 1:** Glacier characteristics.

Hemisphere	South		North	
Location	Deception Island		Kamchatka Peninsula	
Glacier name	Kirkwood	Macaroni	Gorely	Mutnovsky
Sample name	KIR	MAC	GOR	MUT
GPS Coordinates	62°59′56.93″S, 60°38′15.39″W	62°54′27.48″S, 60°32′19.62″W	52°33′27.45″N, 158°02′15.95″E	52°26′57.51″N, 158°11′43.05″E
Altitude (m)	326	480	2,275	2,320
*in situ* Tª (°C)^a^	−0.5	0.0	0.0	0.0
pH	5.5	6.1	7.0	5.5
Volcano activity	Dormant	Dormant	Active	Active
Last eruption (year)	1970	1970	2010	2000

a Temperature and pH are media values of three sampling replicates.

Ice cores (6 cm in diameter and 1 m in length) were recovered using a custom stainless steel crown adaptor on a cordless power drill. The samples for this study were collected from the lower part of the basal glacial fronts, to have an easier access to the subglacial zone. Samples were taken at the base of the glaciers, in the zone of contact with the lower bedrock. For this study 12 samples [four cores: two volcanic glaciers from Antarctica (Macaroni—MAC—and Kirkwood—KIR)], and two volcanic glaciers from Kamchatka (Gorely—GOR—and Mutnovsky—MUT) with three adjacent replicates each (named a, b, c) were obtained. Temperature and pH were *in situ* measured in the ice samples with a calibrated pH meter with SenTix® electrodes (WTW, Weilheim, Germany).

Ice samples were obtained by removing 20–30 cm of thick debris. Samples were wrapped in plastic bags and stored at −20°C until use. Ice samples were processed by using a surface decontamination and melting procedure consistent with previous studies (Bidle et al., 2007; Garcia-Lopez et al., 2021b). The ice core was removed from −20°C. Only the bottom part of each ice core (40 cm) was used. The exterior 1-cm shell of ice samples was ablated. These procedures were effective at removing surface contamination from inner shell ice samples (Rogers et al., 2004; Christner et al., 2005). The decontaminated interior ice was thawed in a sterile plastic bag at 4°C and used for analyses. The meltwater samples were individually filtered through 0.22 μm pore size hydrophilic polycarbonate membranes (Millipore HTTP) using a vacuum pump. Negative pressure used for the filtration of the samples through the membranes was −0.5 bar. To control for laboratory contamination, 500 ml of MilliQ rinse water was frozen, thawed, filtered, and subjected to identical analytical procedures. All procedures were performed by using bleach-sterilized work areas, a UV-irradiated laminar flow hood, ethanol-sterilized tools, and sterilized gloves. The fractions obtained in the filters were used for DNA extraction, and the filtered waters were chemically analyzed.

### Ice Geochemistry

The geochemistry of every ice core sample was characterized using several complementary techniques.

Assays for NH_4_^+^, NO_2_^−^, NO_3_^−^, SO_4_^2−^, soluble reactive phosphorus (SRP), and dissolved organic carbon (DOC; Table 2) were carried out by ion chromatography in an 861 Advance Compact IC system (Metrohm AG, Herisau, Switzerland). Chromatograms were registered with the Metrohm IC Net 2.3 SR6 software. Ions were quantified with internal and external standards prepared from Certified Standard Solutions (TraceCERT®; Merck, Darmstadt, Germany; Garcia-Descalzo et al., 2013).

**Table 2:** Chemical analysis of soluble nutrients in meltwater.

	KIR	MAC	GOR	MUT
NH_4_^+^	2.21 (0.22)	**3.98 (0.33)**	2.11 (0.98)	2.09 (0.87)
NO_2_^−^	2.99 (0.25)	**3.51 (0.41)**	1.97 (0.21)	2.21 (1.01)
NO_3_^−^	2.31 (0.27)	**6.21 (0.33)**	1.24 (2.25)	2.65 (0.98)
SO_4_^2−^	352.58 (22.65)	356.21 (14.28)	478.32 (47.32)	**689.21 (41.32)**
SRP^a^	**0.46 (0.15)**	0.39 (0.22)	0.14 (0.04)	0.38 (0.31)
DOC^b^	45.21 (6.32)	80.21 (9.85)	98.21 (9.32)	**104.25 (10.29)**

Concentrations are expressed as μM (±SEM) of three replicates.

a SRP, soluble reactive phosphorus.

b DOC, dissolved organic carbon.

The highest nutrient values are marked in bold.

The concentration of major (Cl, S, C, K, Na, P, Xe, Mg, Ca, Fe, Co, Mn, Ti, Cr, and Ni), minor, and trace elements (Supplementary Table S1) was quantified by inductively coupled plasma-mass spectrometry (ICP-MS) on a PerkinElmer ELAN9000 ICP-MS quadrupole spectrometer. The samples were introduced into the ICP-MS *via* a RytonTM cross-flow nebulizer (PerkinElmer, Waltham, MA, United States), Scott spray chamber (PerkinElmer), and Cetac ASX-510 autosampler (Omaha, Nebraska, United States; García-Lopez et al., 2021a).

### DNA Extraction and 16S rRNA Gene Library Preparation

The DNA from each 0.22-μm pore filter was extracted and purified with a DNA Isolation PowerWater kit (MO BIO Laboratory, Inc.). Extraction procedures were identical for all samples. DNA concentration was determined using a Nanodrop 2000p (Garcia-Lopez et al., 2021b). Amplicons were sequenced using MiSeq Illumina 2 × 300 bp chemistry 16S rRNA gene amplicon sequencing. The primers of the V3–V4 regions of the 16S rRNA gene (forward sequence CCTACGGGNGGCWGCAG; reverse sequence GACTACHVGGGTATCTAATC) were used to identify bacteria as previously reported (Martinez-Alonso et al., 2019).

### Metabarcoding Data Processing

Quality analyses of reads were performed using FastQ Screen software (version 2; Wingett and Andrews, 2018). Contigs were trimmed to include only the overlapping regions using PANDAseq Assembler (Bartram et al., 2011). This software does also remove the sequence of the primers, discarding the pairs that do not have primer sequences. For the analysis, the QIIME2 software was used. The sequences of all samples were grouped to define the amplicon sequence variants (ASVs). Sequences were aligned against the SILVA (v1.2.11) database.

### Statistical Analysis

Sample diversity was measured with the species richness estimators Observed Species index and Chao 1 index (Chao and Bunge, 2002). The abundance-based coverage estimator (ACE; Chao et al., 2000) and the Shannon (H´) indexes characterize species diversity accounting for abundance and evenness of the species. These indices were calculated using the EstimateS program (version 9.1.0; Colwell, 2013). To find those taxa with significantly different abundance between glaciers, counts were compared.

Statistical analyses were performed using GraphPad Prism version 7.0 (GraphPad Software, La Jolla California United States).^1^

The effects of the chemical composition of glacial ice, as well as the influence of the environment on the microbial community composition, were investigated by multivariate statistical analysis developed with CANOCO version 5 software (Microcomputer Power, Ithaca; Jongman et al., 1995; Ter Braak, 1995). Several gradient analysis methods were used: principal component analysis (PCA), canonical correspondence analysis (CCA), and detrended correspondence analysis (DCA). The parameters used in each analysis are summarized in Supplementary Table S3. Species data were not transformed, except in some specific analysis that is explained in results. The analyses were evaluated with the Monte Carlo test with 500 permutations.

## Results and Discussion

### General Characteristics and Chemical Properties of the Glacial Ice

When comparing these glaciers, the most remarkable geophysical analogies were the low temperature (Table 1) and the high solar radiation (Ruiz-González et al., 2013).

As for the differences, the two Arctic glaciers are located in the interior of the Kamchatka peninsula, and the materials they wash down contribute to the formation of soil and the physical–chemical alteration of the runoff waters. On the contrary, Antarctic glaciers draw into the sea. The Kirkwood glacier empties into the volcanic caldera of Deception Island, while the Macaroni glacier flows into the open sea. These separate locations contribute to some of the differences found in microbial populations. However, the main differences between these groups of glaciers were that the Arctic ones are found on active volcanoes, while the Antarctic ones run on dormant volcanoes. Although volcanoes in Deception Island constantly emit volcanic gases through fumaroles (Baker et al., 1975), the volcanoes of the Kamchatka Peninsula are much more active, with abundant emissions of gases and pyroclastic materials. This fact may introduce a bias in the study. Another important difference, latitude aside, is the altitude (Table 1). The volcanoes considered in Antarctica are located in mountains much lower (326 and 480 m) than those in the north, which exceed 2,000 m in altitude. Another difference observed between the physicochemical characteristics of the meltwater samples was the pH, which was neutral in the Gorely glacier and slightly acidic in the rest of the glaciers (Table 1). Additionally, Antarctic glaciers are more isolated as they are located on an island and are also located at higher latitudes (62°S vs. 52°N; Table 1). Yet the main difference seems to be the remoteness from other continents that leads to geographic insulation, which is increased by the Antarctic Circumpolar Current (Naor et al., 2012). This oceanic stream, the most important in the Southern Ocean, flows clockwise (as seen from the South Pole) and is circumpolar due to the lack of any landmass connecting with Antarctica. This fact keeps the warm waters of the ocean away from Antarctica, which allows the continent to maintain its enormous ice sheet (Donohue et al., 2016). The Kamchatka Peninsula is also subjected to ocean currents such as the East Kamchatka Current (Oyashio or Kurile current), but it is not so cold and has a nutrient-rich nature (Qui, 2001; Jones and Solomina, 2015).

The quantification of nitrogen, sulfur, and phosphorus ions is relevant because these compounds are used by microorganisms as nutrients. Detection limits for these constituents ranged from 0.1 to 2.0 μM. The analyses of NH_4_^+^, NO_2_^−^, NO_3_^−^, SO_4_^2−^, SRP, and DOC in the ice samples (Table 2) demonstrated the existence of a higher concentration of compounds with nitrogen and phosphorus in the Antarctic samples than in the Arctic samples. This result may be due to the presence of detritus from penguins and other marine animals that populate the coast of Deception Island. However, sulfur and carbon compounds were more abundant in Arctic glaciers. It has been described that this type of molecule can be emitted by active volcanoes (Weinbauer et al., 2017).

Of all the chemical elements that were detected in ICP-MS analyses, only those shown in Supplementary Table S1 presented values greater than the detection threshold. Constituents that were below detection limits ranged from 0.3 to 0.7 ppb. The main volcanic materials in these volcanoes are basalt (rich in Mg and Ca, and in alkali oxides of Na and K) and olivine (magnesium iron silicate). These elements were present in all samples and especially in glaciers from active volcanoes. The concentrations of the 10 most abundant elements were taken into account for the studies of interrelationships between bacterial populations and these ion concentrations.

In addition, the samples with the highest concentrations of chlorine and sodium were those from the Antarctic glaciers (Supplementary Table S1). This difference may be due to the proximity of these glaciers to the sea. Another remarkable difference was observed in sulfur concentrations, as had been observed for SO_4_^2−^, since active volcanoes such as those in Kamchatka have been described to emit sulfur as sulfur dioxide or hydrogen sulfide, bromide, carbonyl sulfide, and organic compounds.

### Microbial Community Composition

**Figure 3:**
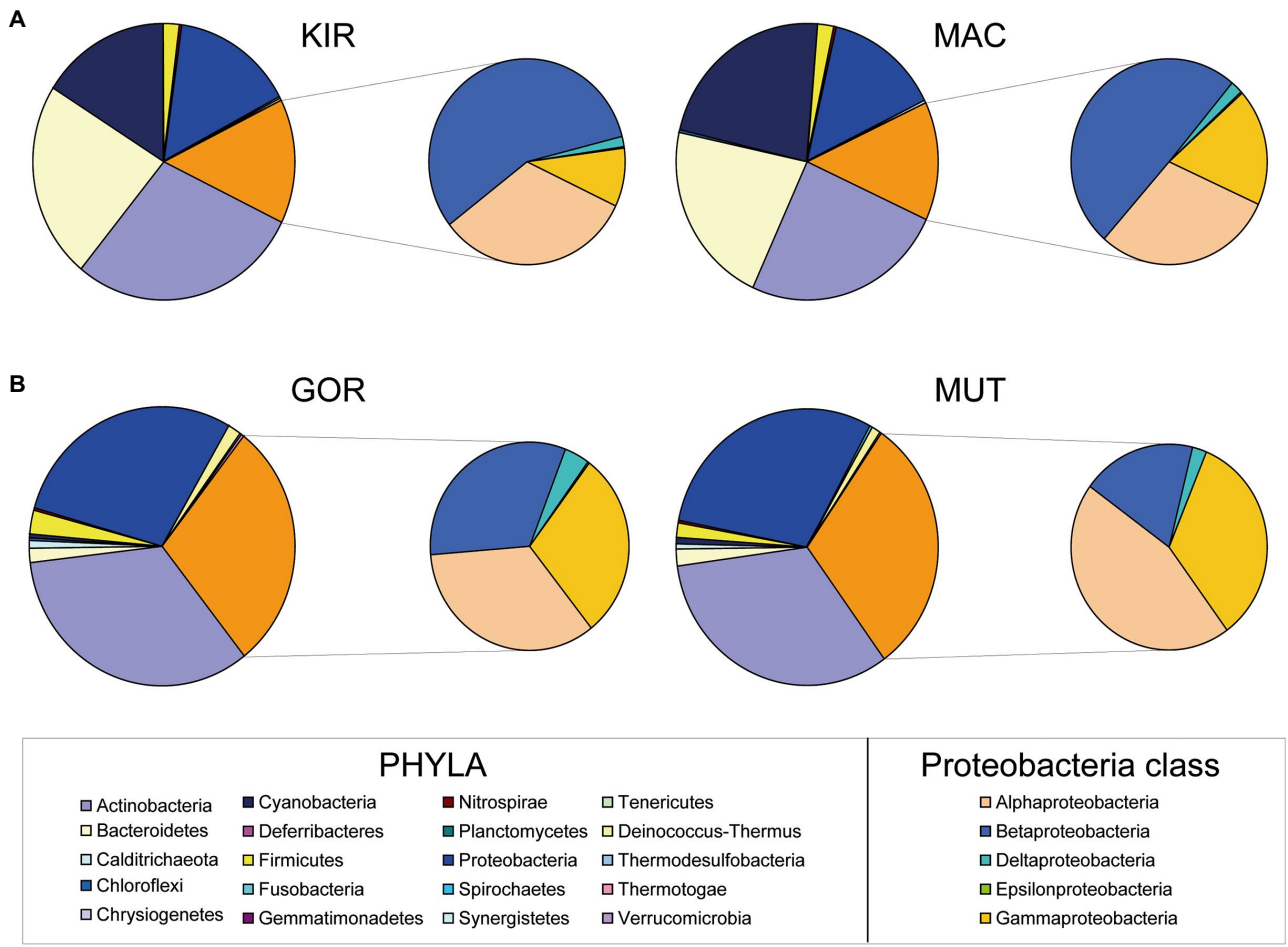
Relative abundances of major phyla of bacteria and of class Proteobacteria. Graphs are based on 16S rRNA gene sequencing data of glacial ice samples.

Amplification and sequencing of the V3 and V4 regions of the 16S rRNA gene rendered a total of 1,004,156 reads which belonged to 1,246 ASVs (Supplementary Table S2) and 23 phyla (Figure 3). Almost all of the diversity of the Arctic bacterial populations was limited to two phyla: Actinobacteria (34%) and Proteobacteria (30%). Among the Proteobacteria, the majority belonged to the classes Alphaproteobacteria (12%) and Gammaproteobacteria (9%; Figure 3A). Diversity in Antarctic populations was much greater. The major phyla were Actinobacteria (27%), Bacteroidetes (23%), Cyanobacteria (20%), and Proteobacteria (15%; Figure 3B). Within the Proteobacteria, the majority belonged to the classes Betaproteobacteria (8%) and Alphaproteobacteria (5%; Figure 3B). It is unknowable from DNA barcodes whether any of these organisms are actively growing or even alive in the glacier. Other techniques will be necessary to clarify this issue.

**Figure 4:**
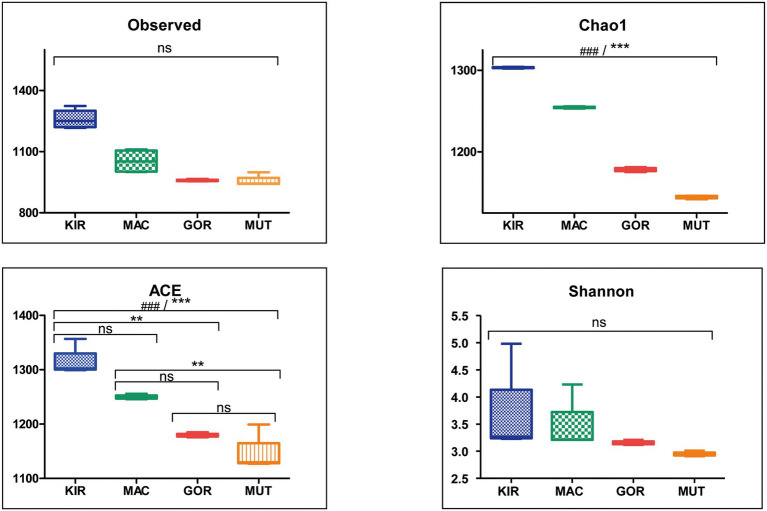
Alpha diversity indexes for microbial populations in volcanic glaciers. Box-plots showing alpha diversity in ice samples using the Observed Species index, the Chao 1 index, the ACE index, and the Shannon index. Statistical differences were studied by ANOVA test (ns, nonsignificant; ^###^*p* < 0.0001). Statistical significance was achieved by Bonferroni’s multiple comparison test (ns, not significant; ^**^p < 0.01; ^***^p < 0.001).

A significant difference between both groups of glaciers was the composition and diversity of microorganisms, which was much higher in Antarctic glaciers (Figure 4). The diversity in the glacier samples was measured using the Observed Species index, the Chao 1 index, the ACE index, and the Shannon index (Figure 4). The greater diversity observed in the Antarctic samples in relation to the Arctic ones stands out.

Regarding microbial populations, several common majority genera were found, such as *Hymenobacter*, *Glaciimonas*, *Actinoallomurus*, *Frankia*, *Pontibacter*, or *Symploca* (Supplementary Figure S1). The most abundant bacterial genera in Antarctic glaciers were *Hymenobacter*, *Pontibacter*, *Frankia*, *Cyanobacterium*, and *Symploca*, while in Arctic glaciers they are *Glaciimonas*, *Actinoallomurus*, *Deinococcus*, *Polaromonas*, and *Methylobacterium*.

All bacterial phyla were represented in all glaciers except Chlorobi, which was only present in KIR samples. There were some exclusive genera of the Arctic glaciers (i.e., *Glaciimonas*, *Thermoleophilum*, *Dinitrobacterium*, and *Nitriliruptor*) and other genera that were only identified in Antarctic glaciers (i.e., *Cyanobacterium*, *Salinibacterium*, *Flavobacterium*, and *Singulisphaera*).

### Microbial Community Distribution

**Figure 5:**
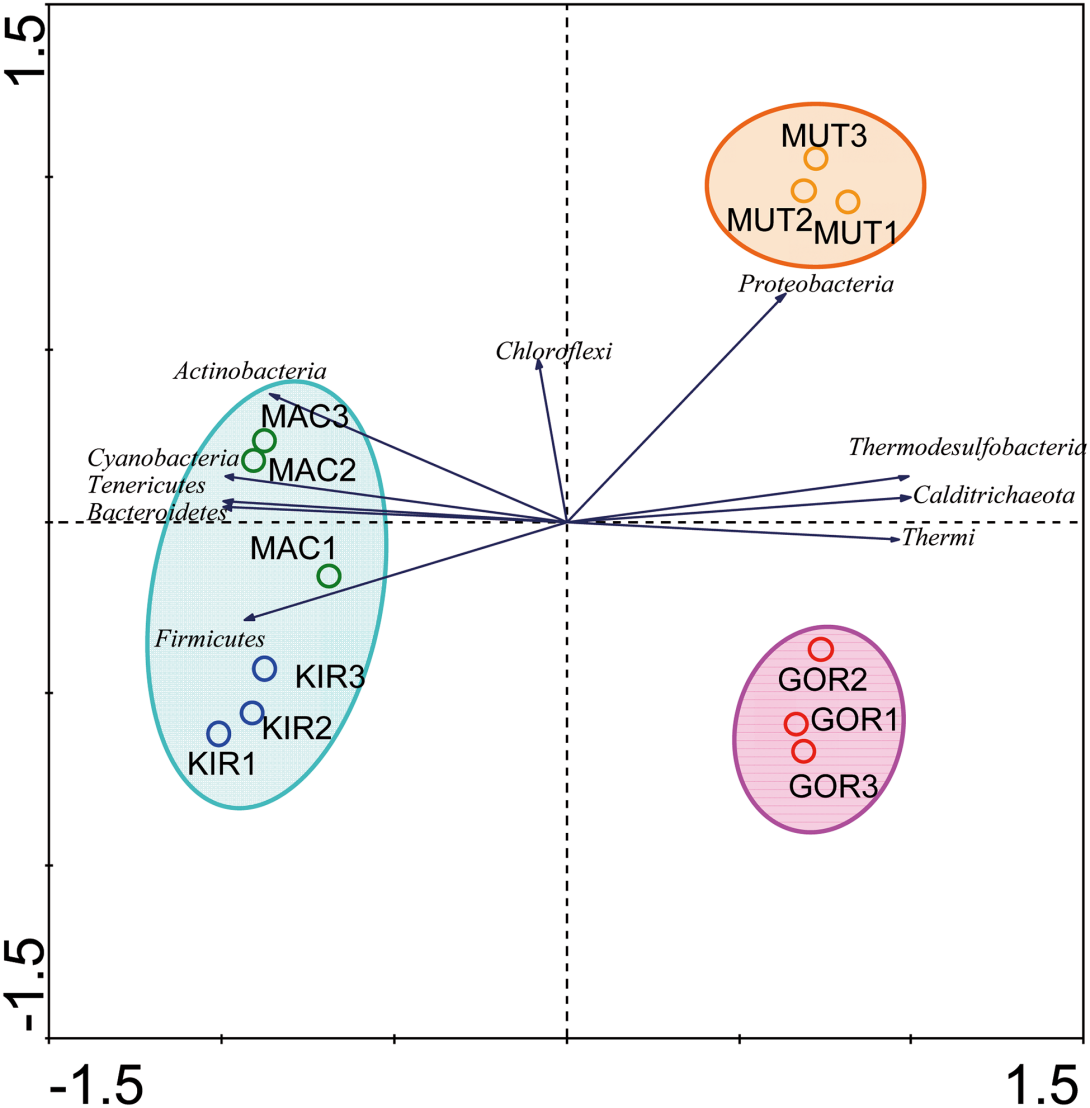
Comparison of the microbial communities using the multivariate statistical analysis principal component analysis (PCA). The diagram display circles representing sampling sites, triangles that represent phyla, and arrows that indicate the direction of increase for the relative abundances.

To determine how the distribution of microbial communities is affected by the geographic and physicochemical properties of glacial ice, several multivariate statistical analyses were carried out. Firstly, a PCA (Supplementary Table S3, analysis no. 1) based on the relative abundances of bacterial phyla was performed to compare how different the microbial composition was in glaciers. In this analysis, the centered log-ratio transformation was used. These results confirmed that microbial communities in glaciers were quite different (Figure 5). The samples MAC and KIR were grouped and associated with an increase in the relative abundance of the phyla Firmicutes, Cyanobacteria, Bacteroidetes, Tenericutes, and Actinobacteria. Aside were the other two populations. The MUT samples were closely associated with a higher relative abundance of Proteobacteria.

**Figure 6:**
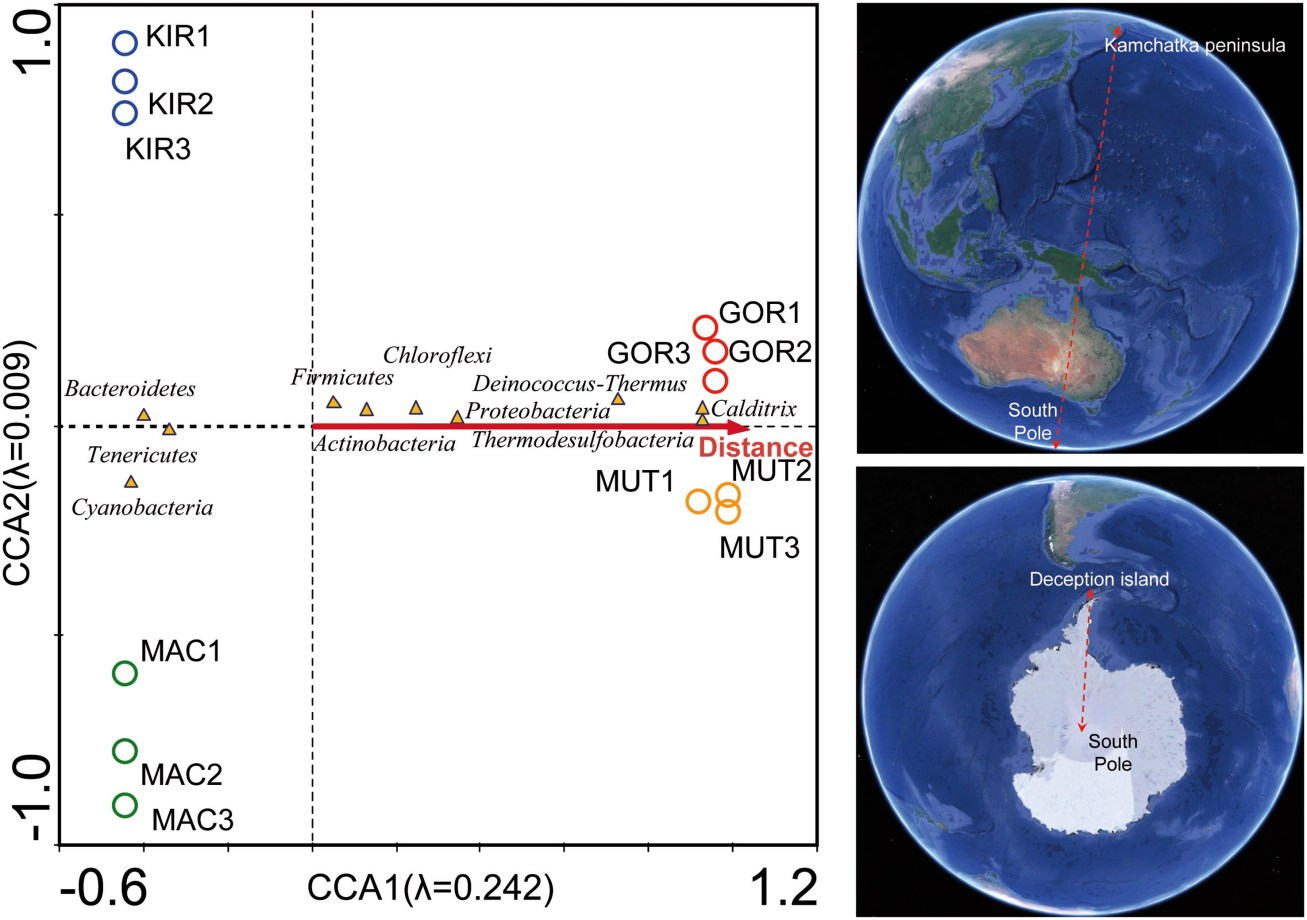
Canonical correspondence analysis (CCA) of the bacterial phyla with respect to the latitude. Analysis of bacterial communities with respect to the distance to the South Pole. The diagram displays triangles that represent taxonomic groups and an arrow that represents the latitude. Map image source: Google Earth Pro.

Regarding their geographical location, some phyla were concentrated in certain latitudes. A DCA analysis showed that there was a distribution gradient of the phyla with respect to the distance from each sampling point to the South Pole (Supplementary Table S3, analysis 2; Figure 6).

**Figure 7:**
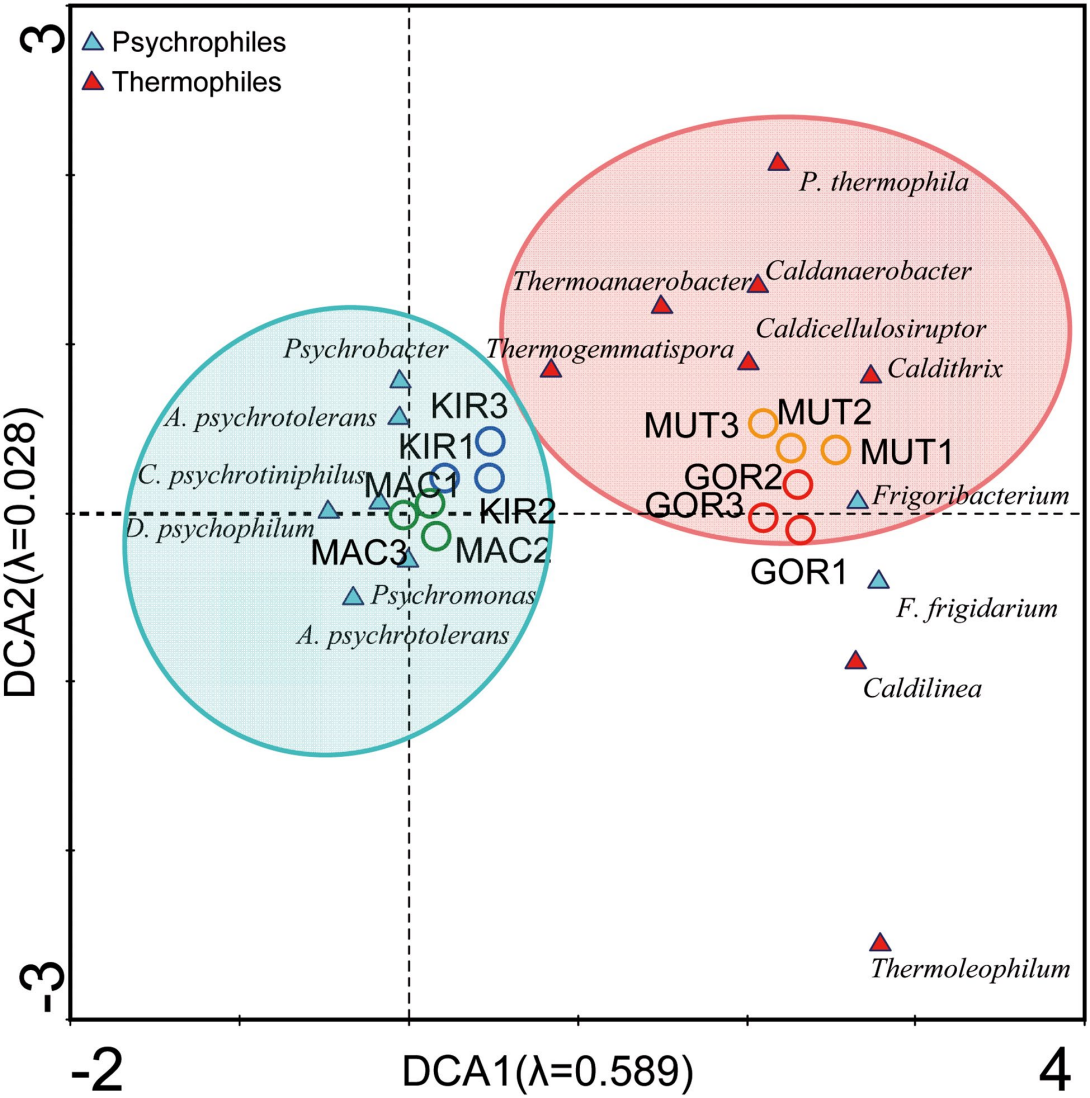
Detrended correspondence analysis (DCA) of the main representants of psychrophiles and thermophiles at the level of genus.

Although temperature measurements were similar in all glaciers (ranged between −0.5 and 0°C; Table 1), a CCA analysis showed a different distribution of the main genus of psychrophilic and thermophilic representants (Supplementary Table S3, analysis 3; Figure 7). This trend may be due to the fact that the tectonic activity is much higher in the Arctic volcanoes than it is in the Antarctic craters. Abundant types of thermophilic bacteria and archaea have also been described in glacial environments near active volcanoes in other volcanic regions such as Iceland (Kelly et al., 2011).

**Figure 8:**
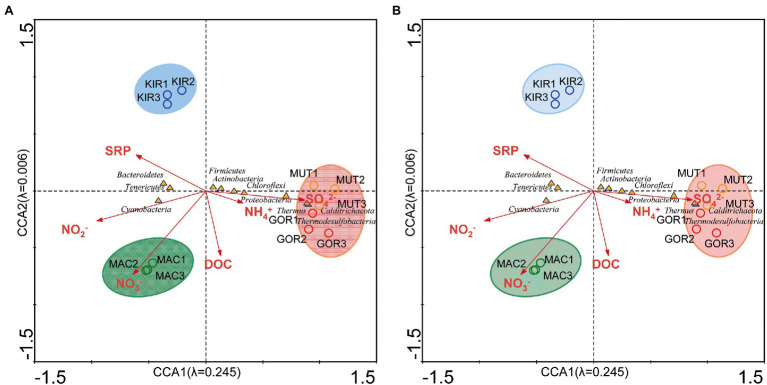
Canonical correspondence analysis of the bacterial phyla with regard to the chemical composition of ice. **(A)** Analysis of bacterial communities with respect to the concentration of NH4^+^, NO^2−^, NO^3−^, SO_4_^2−^, soluble reactive phosphorus (SRP), and dissolved organic carbon (DOC). **(B)** Analysis of bacterial communities with respect to concentration of C, K, Cl, Na, S, Si, Fe, Ca, Mn, and Br. The diagram displays triangles that represent taxonomic groups and arrows that symbolize environmental variables. The axes are scaled in SD units.

Canonical correspondence analysis with the concentration of NH_4_^+^, NO_2_^−^, NO_3_^−^, SO_4_^2−^, SRP, and DOC was used to estimate the proportion of the community variability attributable to these variables. The eigenvalues corresponding to the four ordination axes showed the interrelationships between bacterial phyla and these ion concentrations. This analysis demonstrated that 25.4% of the bacterial variability could be explained by these variables (Supplementary Table S3, analysis 4; Figure 8A). Some phyla of bacteria such as Calditrichaeota, Deinococcus-Thermus, and Thermodesulfobacteria were positively associated with the NH_4_^+^ and SO_4_^2−^ levels.

The CCA with C, K, Cl, Na, S, Si, Ca, Fe, Mn, and Br (Supplementary Table S3, analysis 5; Figure 8B) demonstrated that 25.5% of the bacterial variability could be explained by these ion concentrations. It is noteworthy that chlorine and sodium ions were more associated with samples taken from Antarctic glaciers. As mentioned above, it is possible that this drift is due to the proximity of these glaciers to the sea. Various bacterial species have been reported to attest to the exchange of microorganisms between glaciers and their runoff coastal waters. For example, the genus *Hymenobacter* has been described as a possible sentinel of this transport between glaciers and their downstream seawaters (Garcia-Lopez et al., 2019). In our samples, this genus was much more abundant in the ice samples of the Antarctic glaciers than in the Arctic ones. Other examples of marine microorganisms found in these volcanic glaciers were *Marinobacter* (Button et al., 2004), *Roseospira* (Chakravarthy et al., 2007), and *Polaribacter* (Gosink et al., 1998).

**Figure 9:**
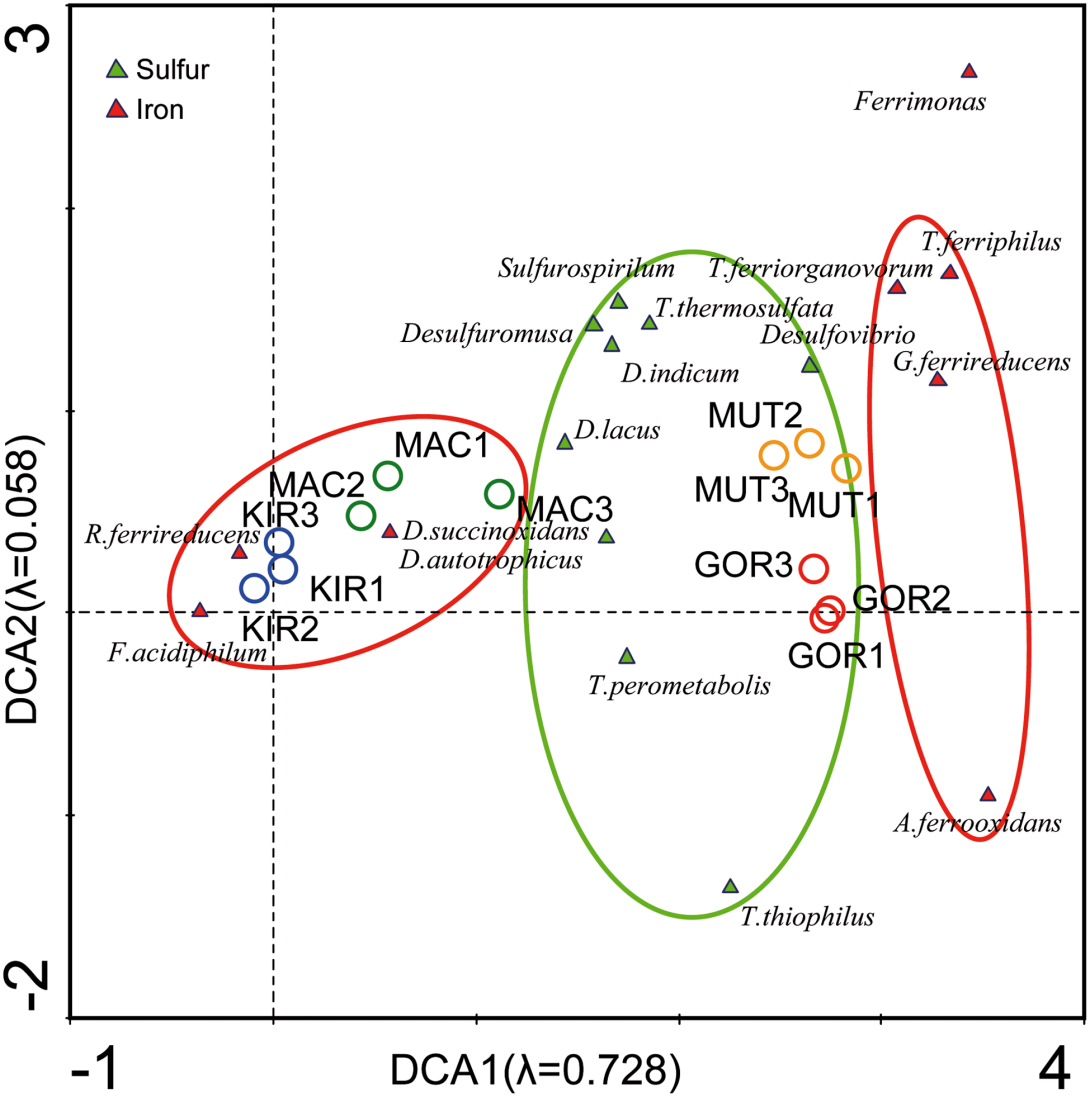
Detrended correspondence analysis of the main representants of iron and sulfur metabolizers at the level of genus.

Moreover, sulfur and iron ions were associated with samples from active volcanoes. Therefore, another DCA was performed at the genus level (Supplementary Table S3, analysis 6; Figure 9). This analysis showed a preferential distribution of sulfur-metabolizing bacterial genera in Arctic volcanoes (i.e., such as *Thiomonas*, *Desulfuromusa*, and *Thermodesulfovibrio*), while iron-metabolizers were mostly distributed in the Antarctic samples (i.e., *Rhodoferax*, *Ferrimicrobium*, and *Deferribacter*), although they appeared throughout the diagram. By applying the contingency Fisher’s exact test, it was obtained that at a confidence level of 95%, there was a significant association (*p* < 0.0001) between the two variables (south–north and sulfur–iron metabolizers).

Finally, a DCA analysis (not shown) was carried out with the main genera of radio-tolerant bacteria such as *Deinococcus*, which is even one of the majority genera of the GOR and MUT glaciers (Supplementary Figure S1). It is well-known that microorganisms that inhabit glaciers have a high tolerance to ultraviolet radiation (Singh and Gabani, 2011; Madigan et al., 2012). However, these results were not significant, since the distribution of radio-tolerant bacteria was equivalent in the ice samples from both hemispheres.

### Main Differences Between Microbial Communities Inferred From Taxonomy

**Figure 10:**
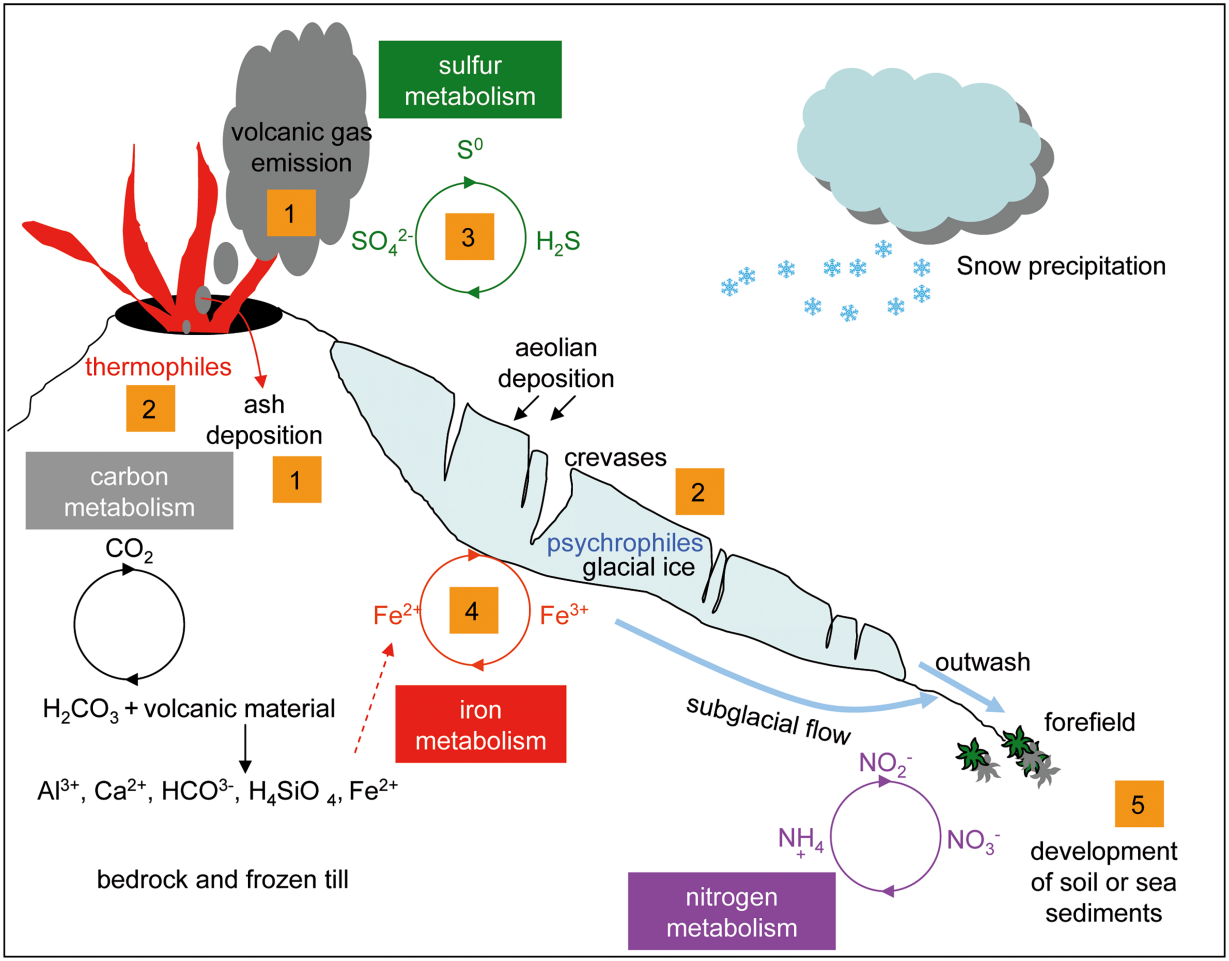
Model of the analogies and differences in the microbial populations of volcanic glaciers. (1): gas emission and pyroclastic material deposition. (2): thermophiles and psychrophiles balance. (3): microorganisms involved in the sulfur cycle. (4): microorganisms involved in the iron cycle. (5): development of soil and mudflow deposits in the sea coast.

The main differences when comparing these four glaciers were: (i) their different volcanic gas emission and ash deposition; (ii) the thermophiles–psychrophiles balance; (iii) the microbial participants in sulfur and (iv) iron cycles; and (v) the development of soil or contribution to the sea sediments (Figure 10).

All these glaciers contain black layers rich in ash and pyroclastic material embedded in the glacial ice. Microscopic organisms carried by the wind together with ashes can extend the geographical range of live colonies (Van Eaton et al., 2013). These materials constitute an essential source of nutrients to be used by the microbial communities. Conversely, microorganisms contribute to the weathering of volcanic rocks and to the exchange of ions between the pyroclastic material and the meltwater of glaciers. The significant amounts of reduced iron and manganese present in basaltic rocks provide potential energy sources for chemolithotrophic bacteria. Some bacterial species such as the acidophilic iron-oxidizing *Ferrimicrobium acidiphilum* (Actinobacteria) and the iron-reducers *Rhodoferax ferrireducens* (Betaproteobacteria), and *Deferribacter autotrophicus* (Deferribacteres) were closely associated with the Antarctic glaciers (Aytar et al., 2015; Garcia-Lopez et al., 2019; Slobodkin et al., 2019).

Considering that some of the main components of volcanic gases are water vapor and sulfur, either as sulfur dioxide (at high temperature) or hydrogen sulfide (at low temperature; Woitischek et al., 2020), it could be explained that the microorganisms that participate in the sulfur cycle, such as *Thiomonas thermosulfata*, *Thermodesulfovibrio thiophilus*, and *Desulfovibrio*, were found mainly associated with ice samples from Arctic glaciers, which are more active than Antarctic glaciers.

Both chemolithotrophs that synthesize organic compounds by fixing carbon dioxide and chemoorganotrophs that degrade organic matter, participate in the carbon cycle. The organic compounds, carbon monoxide, and carbon dioxide metabolized by microorganisms come from volcanic gases, which in this case are much more profusely emitted by the volcanoes of Kamchatka than those of Deception Island. The organic matter may also come from plant and animal remains contained within ice. Methane is one of the main gaseous components released by volcanoes, and the metabolism of this compound is very relevant in volcanic environments in which a great diversity of archaeal and bacterial communities has been identified (Cheng et al., 2012). In our samples, several methylotrophs were found (i.e., *Methylibium*, *Methylobacterium*, and *Methylotenera*), which can catabolize methane and many other carbon compounds. For example, *Methylotenera* exclusively uses methylamine as a single source of energy, carbon, and nitrogen (Kalyuzhnaya et al., 2006).

## Conclusion

In summary, the main contrasts found in this study among these glaciers were the differences in the composition and diversity of microbial populations and the existence of taxonomic groups exclusively found exclusively in one of the hemispheres, such as the phylum Chlorobi. However, some factors had a special influence on the results of this research, such as the different volcanic activity, which may introduce a bias in the conclusions about the distribution of thermophiles and psychrophiles or of the iron and sulfur metabolizers. Therefore, future study of glaciers in volcanic regions will allow us to confirm these results and broaden our knowledge on the microbial populations that inhabit volcanic glaciers. To know whether there is microbial growth in this subglacial ecosystem or whether the microorganisms are dead or dormant will be other interesting question for future research. The results obtained in this study deepen the better understanding of a regional diversity in the volcanic glaciers and will have application in a variety of disciplines such as microbial ecology, climate change studies, biotechnology, astrobiology, and space exploration.

## Data Availability Statement

The sequences obtained by 16S rRNA sequencing were deposited in NCBI BioProject PRJNA777768, Short Read Archive (SRA SRS10910775-SRS10910778).

## Author Contributions

CC conceived and planned the experiments, performed field sampling, and managed the acquisition of funds. FR-B, EG-L, SS-C, SP, and MM-C performed laboratory work and data acquisition. CC, SS-C, SP, and MM-C wrote, revised, and edited the manuscript. All authors have read and agreed to the published version of the manuscript.

## Funding

This research has been funded by grant nos. PID2019-104205GB-C22 and MDM-2017-0737 Unidad de Excelencia “Maria de Maeztu”-Centro de Astrobiología (INTA-CSIC) by the Spanish Ministry of Science and Innovation/State Agency of Research MCIN/AEI/10.13039/501100011033. EG-L is supported by PTA2016-12325-I grant provided by MCIN/AEI/10.13039/501100011033.

## Conflict of Interest

The authors declare that the research was conducted in the absence of any commercial or financial relationships that could be construed as a potential conflict of interest.

## Publisher’s Note

All claims expressed in this article are solely those of the authors and do not necessarily represent those of their affiliated organizations, or those of the publisher, the editors and the reviewers. Any product that may be evaluated in this article, or claim that may be made by its manufacturer, is not guaranteed or endorsed by the publisher.

## Footnotes

^1^ www.graphpad.com

## Abbreviations

CCA: Canonical correspondence analysis
DCA: Detrended correspondence analysis
DOC: Dissolved organic carbon
GOR: Gorely glacier
ICP-MS: Inductively coupled plasma-mass spectrometry
KIR: Kirkwood glacier
MAC: Macaroni glacier
MUT: Mutnovsky glacier
PCA: Principal component analysis
SRP: Soluble reactive phosphorus
