# Supplementary material for: Microbial Communities in Volcanic Glacier Ecosystems
Source: Front Microbiol. 2022 Apr 25;13:825632. doi: 10.3389/fmicb.2022.825632 (PMC9084427; doi:10.3389/fmicb.2022.825632)

Supplementary Material

Comparison of the microbial communities in the volcanic glaciers of the South and North hemispheres

*Eva Garcia-Lopez, Fatima Ruiz-Blas, Silvia Sanchez-Casanova, Sonia Peña Perez, Maria Luisa Martin-Cerezo and Cristina Cid**

* Corresponding Author: cidsc@inta.es

This file includes:

I. Supplementary Tables S1-S3

II. Supplementary Figures

I. Supplementary Tables S1-S3

Table S1. Chemical analysis of ions in meltwater. Concentrations are expressed in ppb (±SEM) of three replicates. [a]BD: below detection.

File: Table S1.xlsx

Table S2. Analysis of bacterial 16S rRNA genes retrieved from ice samples. OTU level aggregate counts of 3 sampling replicates.

File: Table S2.xlsx

Table S3. Correspondence analysis and Species-environment correlations (λ).

No. of analysis	Type of analysis	Environmental variables	λ_1	λ_2	λ_3	λ_4	Figure
1	PCA	-	0.983	0.012	0.004	0.001	5
2	CCA	Distance to South Pole	0.242	0.009	0.003	0.001	6
3	DCA	Adaptation to temperature	0.589	0.028	0.001	0.000	7
4	CCA	NH_4^+, NO_2^-, NO_3^-, SO_4^{2-}, SRP, DOC	0.245	0.006	0.003	0.000	8A
5	CCA	Mg, Fe, Ca, Mn, K, C, Si, Na, Cl, S, Br, Co, Cu	0.246	0.006	0.003	0.000	8B
6	DCA	Sulfur and iron metabolism	0.728	0.058	0.001	0.000	9

II. Supplementary Figures

Figure S1. Graphic representation of the relative abundance (No. reads) belonging to the most abundant genus in each glacier.

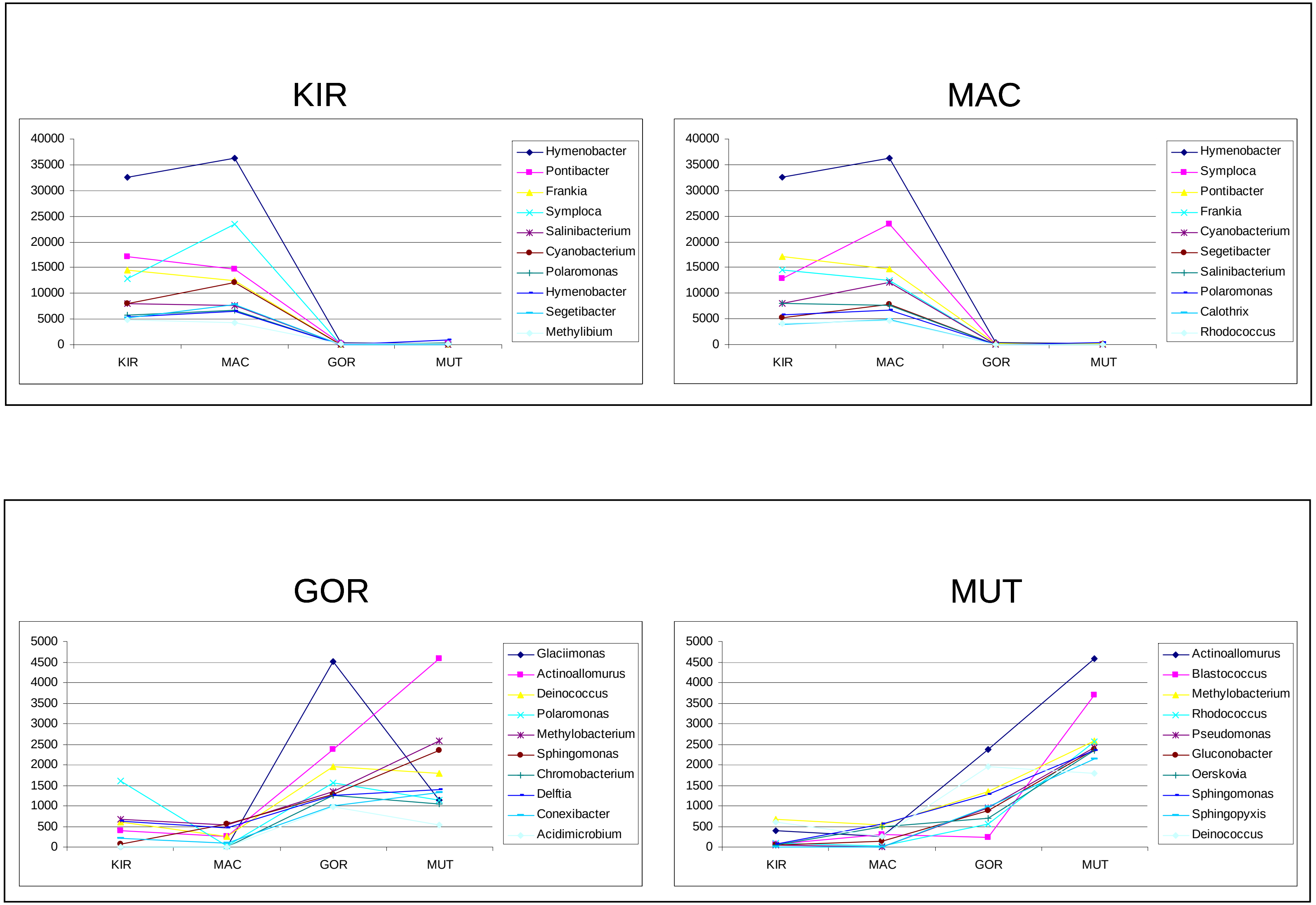

Supplement: Supplementary file 3 [file Data_Sheet_1.pdf]
